# Burden of immune-related skin diseases worldwide, 1991–2021: insights and prediction from the Global Burden of Disease Study

**DOI:** 10.3389/fimmu.2025.1668840

**Published:** 2025-11-11

**Authors:** Min Leng, Ping Qi, Ronghua Li, Feiyu Gong, Zairong Wei

**Affiliations:** 1 Department of Burns and Plastic Surgery, Affiliated Hospital of Zunyi Medical University, Zunyi, China; 2 Department of Burns and Plastic, Dazhou Central Hospital, Dazhou, China; 3 The 2011 Collaborative Innovation Center of Tissue Damage Repair and Regeneration Medicine, Affiliated Hospital of Zunyi Medical University, Zunyi, China; 4 The Collaborative Innovation Center of Tissue Damage Repair and Regeneration Medicine, Zunyi Medical University, Zunyi, China

**Keywords:** immune-related skin diseases, Global Burden of Disease, epidemiology, prevalence, dermatitis, urticaria, alopecia areata, psoriasis

## Abstract

**Background:**

Immune-mediated dermatological conditions, including dermatitis, urticaria, alopecia areata, and psoriasis, are common skin diseases that contribute to substantial health loss, economic burden, and pain across individuals of all ages worldwide.

**Methods:**

Using data from the Global Burden of Disease (GBD) 2021 study, we analyzed age-standardized incidence, prevalence rate, and disability-adjusted life years (DALYs) for global main four immune-related skin diseases—including dermatitis (atopic, contact, and seborrheic), urticaria, alopecia areata, and psoriasis from 1991 to 2021, with corresponding 95% uncertainty intervals (UIs), stratified by sex, age, geographical location, and sociodemographic index (SDI). We further projected incidence through 2035 using a Holt-damped model incorporating trend components but excluding seasonality.

**​Results​:**

Dermatitis had the highest estimated age-standardized prevalence rate (ASPR: 5459.07 per 100,000; 95% UI: 5064.87–5875.73), followed by psoriasis (354.07; 95% UI: 342.42–364.08), urticaria (1094.59; 95% UI: 969.18–1240.42), and alopecia areata (42.89; 95% UI: 41.74–44.14). Immune-related dermatoses consistently showed higher age-standardized rates in females than males. The estimated annual percentage change (EAPC) revealed distinct temporal patterns: dermatitis (-0.155) and alopecia areata (-0.127) showed slight declines, whereas psoriasis exhibited an upward trend (0.24), and urticaria remained stable with a modest increase (0.01). Age distribution: Dermatitis/urticaria peaked in children, alopecia areata in adulthood, and psoriasis in middle age.

**Conclusions​:**

Immune-related skin diseases—including dermatitis, urticaria, alopecia areata, and psoriasis—are highly prevalent worldwide, with notable variations by age, sex, and region. Females are disproportionately affected. These trends underscore the need for targeted, sex- and region-specific public health interventions to optimize the allocation of healthcare.

## 1 Introduction

Immune-related skin diseases—including dermatitis, urticaria, alopecia areata, and psoriasis—are prevalent and contribute substantially to the GBD (1, 2) and represent a diverse group of disorders characterized by aberrant immune responses targeting the skin (3–5). Though not always life-threatening, these disorders can result in persistent physical symptoms and psychological distress, even after clinical remission (6). Their long-term impact affects quality of life and imposes a notable burden on caregivers, health systems, and society at large. The prevalence and distribution of dermatological conditions have been explored in various geographic contexts. These studies underscore the importance of recognizing immune-related skin disease as part of a broader autoimmune and inflammatory spectrum. With the rise in the SDI, multiple studies have shown that environmental and lifestyle transitions—such as gut microbiota dysbiosis, excessive hygiene leading to reduced microbial exposure (the “hygiene hypothesis”), urbanization, and dietary changes—may contribute to the increasing incidence of immune-related skin diseases (7–9). These disorders share several common influencing indicators, including genetic susceptibility, immune dysregulation, psychosocial stress, and environmental pollutants, all of which can disrupt immune homeostasis and damage the skin barrier. Therefore, understanding the shared epidemiological patterns and trends of these diseases is essential to developing integrated prevention and public health strategies.

During the COVID-19 pandemic in Turkey, notable shifts in dermatologic disease patterns were observed, potentially reflecting the impact of altered environmental or societal factors on disease epidemiology (10). Alopecia areata is primarily characterized as an autoimmune disorder involving T-cell-mediated attack on hair follicles, leading to non-scarring hair loss (11, 12). A focus on alopecia areata’s epidemiology is provided by Augustin (13), who was analyzed longitudinal claims data in Germany. Their findings highlight the population-wide prevalence and associated comorbidities, emphasizing the autoimmune nature of alopecia areata and its frequent coexistence with other systemic conditions. Alopecia areata is part of a broader autoimmune and inflammatory spectrum. A systematic review has examined the inflammatory and autoimmune aspects of urticaria, including the potential anti-inflammatory effects of antidepressants and possible associations with mood disorders (14).

This connection supports the biopsychosocial model of dermatologic disease epidemiology, which emphasizes the interplay of biological, psychological, and social factors in disease development (15). Psoriasis, a chronic inflammatory skin disease, is also associated with systemic comorbidities. Psoriasis, along with other skin diseases, involves autoimmune and inflammatory mechanisms, highlighting the need for targeted therapies and biomarker development to address unmet medical needs (16).

Overall, the literature underscores that immune dermatologic conditions such as dermatitis, urticaria, alopecia areata, and psoriasis are prevalent across populations and frequently coexist with both physical and mental health disorders (17). Accurate epidemiological data on immune-related skin diseases are essential for guiding health policy, allocating resources, and designing prevention strategies. Yet, most previous research has focused narrowly on specific conditions or regions, leaving a gap in comprehensive, cross-national comparisons (18, 19). To address this, updated global data on the geographic and temporal trends of these diseases are urgently needed. Such insights are critical to improving disease monitoring, optimizing interventions, and reducing unnecessary healthcare costs.

This study draws on prevalence, DALYs, and incidence for immune-related diseases, disaggregated by location, sex, and age (20). Using the latest data from the GBD study, we conducted a comprehensive analysis of immune-related skin diseases across 204 countries from 1991 to 2021, aiming to provide evidence-based guidance for public health policy development in this field.

## 2 Method

Annual case counts and ASR of incidence, prevalence, and DALYs for immune-related skin diseases, including dermatitis, urticaria, alopecia areata, and psoriasis, were obtained for the period 1991–2021. According to the Global Burden of Disease (GBD) database classification, the term “dermatitis” in this study includes the following categories: atopic dermatitis (ICD-10: L20), contact dermatitis (L23), and seborrheic dermatitis (L21). In addition, urticaria corresponds to ICD-10 code L50, alopecia areata to L63, and psoriasis to L40. Prevalence was selected as the primary measure for analysis because it most accurately reflects the patient burden, provides stable estimates over time, and allows for reliable cross-regional comparisons. All data were sourced from the GBD study, coordinated by the Institute for Health Metrics and Evaluation. The GBD offers comprehensive, standardized estimates of disease, injury, and risk factor burdens across countries and time periods, and serves as a critical resource for global health policy and planning (21).

This study included data from 204 countries and territories, categorized into five SDI levels—ranging from low to high—and geographically grouped into 21 regions, including high-income Asia Pacific and Central Asia (22). SDI, scaled from 0 to 1, integrates per capita income, education, and fertility. Based on GBD 2021, 204 countries were stratified into five SDI levels. This allows comparison of immune-related skin disease burden across socioeconomic levels and assessment of its association with global development disparities.

We extracted annual data on prevalence, incidence, and DALYs across global, regional, and national levels. Prevalence and mortality were expressed per 100,000 population (23). SDI, ranging from 0 to 1, was derived from total fertility rate, income per capita, and mean years of education among those aged 15 and above, with higher values indicating greater development (24). This analysis adhered to the Guidelines for Accurate and Transparent Health Estimates Reporting for cross-sectional studies (25). Given the low fatality of immune-related skin diseases, GBD does not report its mortality estimates; instead, the age-standardized prevalence rate (ASPR) serves as the primary metric for burden assessment, as these conditions contribute little to years of life lost (YLLs). To capture both new cases and non-fatal health loss, we also calculated age-standardized incidence (ASIR) and DALY rates, with the latter integrating fatal and non-fatal losses to summarize total population health gaps.

All results are presented per 100,000 population, accompanied by 95% uncertainty intervals (UIs). These intervals were derived from the 2.5th and 97.5th percentiles of 1,000 draws from the posterior distribution generated by the Bayesian meta-regression model (DisMod-MR 2.1). In the Global Burden of Disease Study 2019 (GBD 2019), 95% uncertainty intervals (UIs) were calculated for each metric to represent the uncertainty surrounding the estimates. These UIs were defined by the 2.5th and 97.5th percentiles of 1,000 ranked values (20).

To analyze temporal trends in immune-related skin diseases, Time series forecasting was performed using the Holt’s damped trend exponential smoothing (ETS(A,Ad,N)) model, which extends the classical Holt linear trend model by incorporating a damping parameter to prevent the trend from extrapolating indefinitely. This model assumes additive errors, additive damped trend components, and no seasonality. Model parameters, including the smoothing coefficients ( α, $\beta^{*}$) and the damping factor ( Φ), were estimated by minimizing the sum of squared errors through maximum likelihood estimation. Model selection was based on the Akaike information criterion (AIC) and Bayesian information criterion (BIC), while predictive performance was evaluated using mean absolute percentage error (MAPE) and root mean square error (RMSE) (26, 27). This approach has been widely adopted for short- to medium-term forecasting due to its stability and interpretability to predict future trends from 2022-2035.

We analyzed ASR of incidence, prevalence, and DALYs using Joinpoint regression to calculate EAPC and assess temporal trends (1991–2021) (28). Sensitivity analyses were conducted to assess the robustness of temporal trend estimates derived from Joinpoint regression. Models with 3 joinpoints were compared using the Bayesian Information Criterion (BIC) and permutation tests (p< 0.05). All analyses were performed using R software, version 4.2.2, with two-sided p< 0.05 indicating a statistically significant difference.

## 3 Results

### 3.1 General status of the global immune-related skin diseases burden

Globally, the age-standardized prevalence rates of major inflammatory and immune-related skin diseases displayed significant regional variation in 2021. Dermatitis bore the greatest burden among the four conditions, with a global ASPR of 5459.07 (95% UI: 5064.87–5875.73) per 100,000 population, followed by urticaria at 1094.59 (969.18–1240.42), psoriasis at 354.07 (342.42–364.08), and alopecia areata at 42.89 (41.74–44.14) in **Table 1**. Across all regions, dermatitis consistently represented the largest proportion of the disease burden, highlighting its widespread public health impact. In contrast, alopecia areata showed the lowest prevalence overall, albeit with modest regional variations.

**Table 1:** Global Age-Standardized Prevalence Rate (ASPR) of immune-related skin diseases in 2021.

Location_name	Alopecia areata	Dermatitis	Psoriasis	Urticaria
	Prevalence (95% UI) Rate	Prevalence (95% UI)) Rate	Prevalence (95% UI)) Rate	Prevalence (95% UI)) Rate
Global	42.89(41.74,44.14)	5459.07(5064.87,5875.73)	354.07(342.42,364.08)	1094.59(969.18,1240.42)
High-income Asia Pacific	63.10(61.17,65.10)	4209.22(3836.45,4644.40)	472.53(455.26,486.55)	1018.42(908.05,1158.22)
High-income North America	80.20(78.34,82.29)	3502.78(3233.12,3824.56)	477.11(463.09,489.63)	1225.38(1153.01,1308.97)
High-middle SDI	38.62(37.49,39.78)	5968.74(5579.48,6403.08)	347.48(335.80,357.32)	1110.69(980.56,1259.99)
Low SDI	35.95(34.99,37.05)	4294.77(3910.94,4718.93)	250.20(241.54,257.59)	1071.99(943.76,1222.86)
Low-middle SDI	38.55(37.44,39.72)	4944.52(4551.30,5385.85)	291.72(282.09,300.15)	1057.81(932.35,1208.14)
Middle SDI	38.73(37.61,39.93)	7466.29(7012.06,7940.58)	326.27(315.21,335.66)	1141.74(1012.99,1298.98)
Andean Latin America	34.78(33.73,35.95)	5336.42(4942.09,5755.75)	333.26(320.84,343.76)	1107.37(976.86,1267.07)
Australasia	63.22(61.34,65.31)	4029.00(3667.53,4423.12)	455.49(437.80,470.60)	993.78(888.25,1121.53)
Caribbean	34.78(33.73,35.95)	6141.31(5750.62,6594.62)	324.76(312.83,335.28)	1108.25(977.70,1267.98)
Central Asia	55.66(54.18,57.46)	4225.42(3874.32,4581.96)	414.42(400.83,426.18)	1196.88(1058.90,1359.85)
Central Europe	55.45(53.99,56.83)	3688.26(3408.60,3997.57)	493.41(480.84,503.12)	1220.54(1110.03,1357.17)
Central Latin America	34.85(33.81,35.90)	4158.12(3774.17,4602.04)	329.50(317.49,339.44)	1139.93(1009.35,1295.49)
Central Sub-Saharan Africa	31.96(30.96,33.03)	3640.09(3272.22,4034.04)	240.02(230.55,248.12)	1109.33(978.74,1269.04)
East Asia	40.21(39.01,41.52)	7394.70(6991.97,7907.22)	360.03(347.71,370.59)	1149.59(1026.65,1300.70)
Eastern Europe	56.48(54.85,58.10)	4579.57(4211.97,4975.09)	495.97(480.51,510.12)	1261.88(1115.81,1433.81)
Eastern Sub-Saharan Africa	31.25(30.38,32.23)	3882.17(3497.59,4319.28)	238.61(230.45,245.56)	1131.05(998.90,1287.94)
North Africa and Middle East	33.16(32.20,34.25)	9668.05(9095 .32,10219.14)	332.29(320.72,342.58)	1057.65(937.75,1201.63)
Oceania	40.00(38.81,41.31)	7364.46(6845.21,7891.86)	257.04(247.70,265.58)	1136.13(1005.65,1297.27)
South Asia	40.20(38.98,41.49)	4383.59(3992.76,4830.51)	298.40(288.65,306.68)	1001.32(879.23,1147.41)
Southeast Asia	40.13(38.96,41.41)	7496.73(7031.55,7998.58)	325.95(315.00,335.19)	1177.85(1045.52,1348.49)
Southern Latin America	63.64(61.64,65.83)	4000.13(3648.72,4410.23)	410.11(395.04,423.60)	987.14(882.48,1111.39)
Southern Sub-Saharan Africa	32.07(31.05,33.09)	3809.12(3421.18,4251.27)	331.11(319.38,340.42)	1154.03(1025.93,1308.77)
Tropical Latin America	36.21(35.17,37.32)	6478.51(6075.41,6948.37)	340.60(329.39,350.08)	1167.17(1037.66,1324.92)
Western Europe	57.77(56.07,59.57)	4284.17(3918.05,4683.81)	465.43(448.59,480.28)	785.42(703.96,884.35)
Western Sub-Saharan Africa	33.37(32.47,34.45)	3619.54(3253.41,4022.71)	252.15(243.95,259.55)	1139.30(1008.25,1295.40)

### 3.2 Immune-related skin diseases in different regions

**Figure 1:**
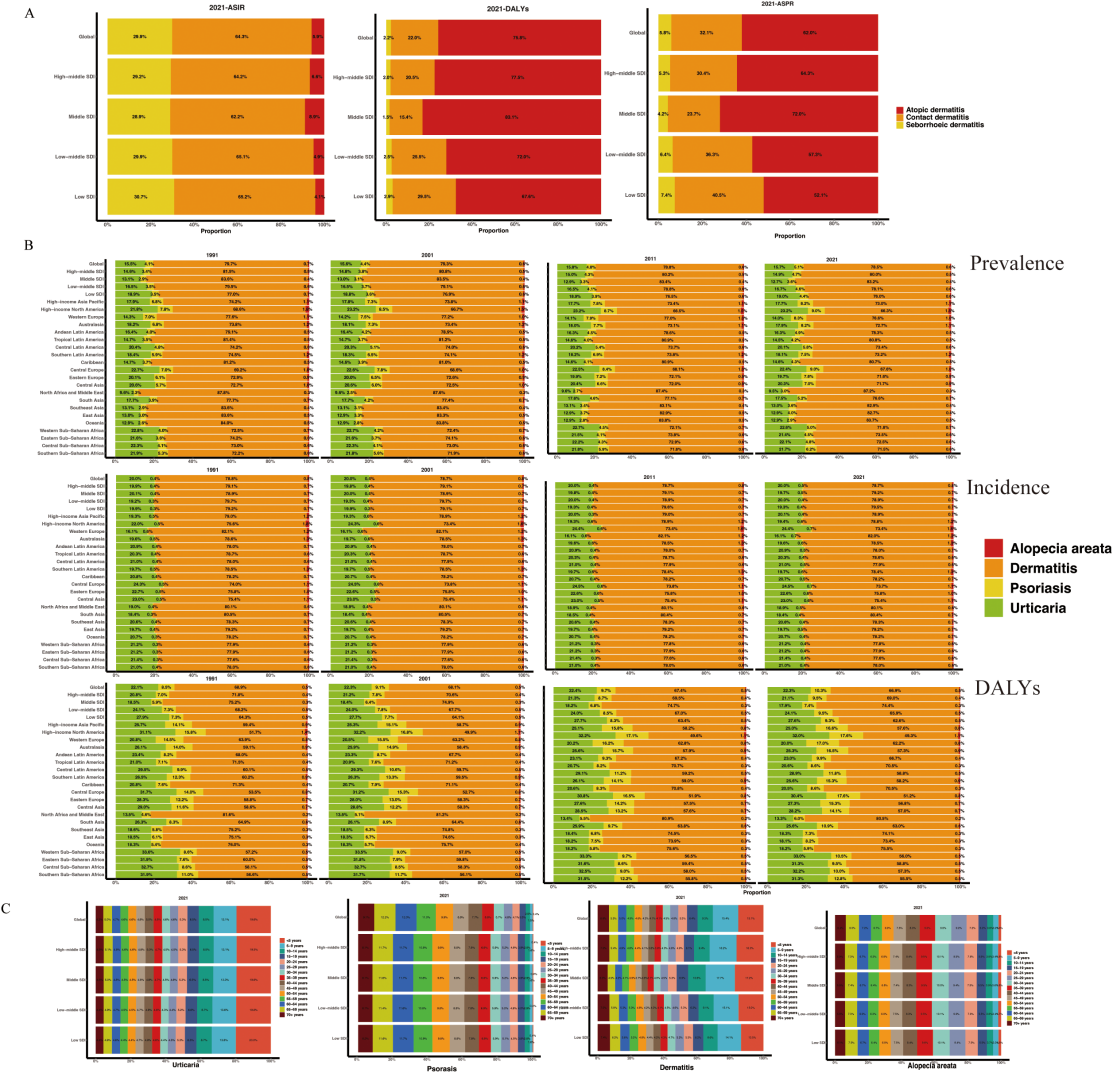
Proportion of dermatitis subtypes **(A)** and immune-related skin diseases **(B)** across 27 territories in ASPR. **(C)** Age-specific distribution of diseases by SDI region in 2021. ASPR, age-standardized prevalence rate; ASR, age-standardized rate; SDI, socio-demographic index.

In the global composition of dermatitis incidence for 2021, contact dermatitis accounted for the largest proportion (64.3%), followed by seborrheic dermatitis (29.9%) and atopic dermatitis (5.9%). This distribution pattern was consistent across all SDI regions in **Figure 1A**. However, atopic dermatitis (75.8%) constituted the major contributor to the global dermatitis DALYs burden, followed by contact dermatitis (22.0%) and seborrheic dermatitis (2.2%).

Based on data from 1991, 2001, 2011, and 2021, the following epidemiological trends were observed regarding immune-mediated skin diseases (**Figure 1B**, **Supplementary Table S1**): Dermatitis comprised the largest proportion of global skin disease incidence, consistently representing the most common immune-mediated dermatological condition. Its relative prevalence remained stable across the three decades, accounting for approximately 80% of total cases. Urticaria was the second most prevalent condition, representing approximately 20% of cases. Alopecia areata and psoriasis demonstrated lower prevalence rates, with psoriasis consistently accounting for the smallest proportion (<1%) of the global disease burden. Prevalence rates generally mirrored incidence patterns across major global regions, showing no significant fluctuations. When examining DALYs, dermatitis remained the leading contributor to the overall burden of skin disease. However, a notable discrepancy was observed for psoriasis, which accounted for a substantially higher proportion of total DALYs (approximately 9-12%) relative to its incidence. This disparity suggests a disproportionately greater long-term disabling impact per case of psoriasis, likely reflecting its chronic nature, associated comorbidities, and significant effects on quality of life that are not fully captured by incidence metrics alone.

**Figure 1C** illustrates age-specific prevalence patterns for immune-mediated skin diseases. Urticaria exhibits a peak prevalence in early childhood, followed by a decline with increasing age. Dermatitis is most prevalent in children under 10 years of age, with a gradual reduction thereafter. Alopecia areata and psoriasis demonstrate similar age-related trends, characterized by an increase in prevalence during early adulthood, a peak in midlife, and a subsequent decrease in later years. These patterns are consistently observed across all SDI regions.

### 3.3 Sex-related differences and EAPC in immune-related skin diseases

**Figure 2:**
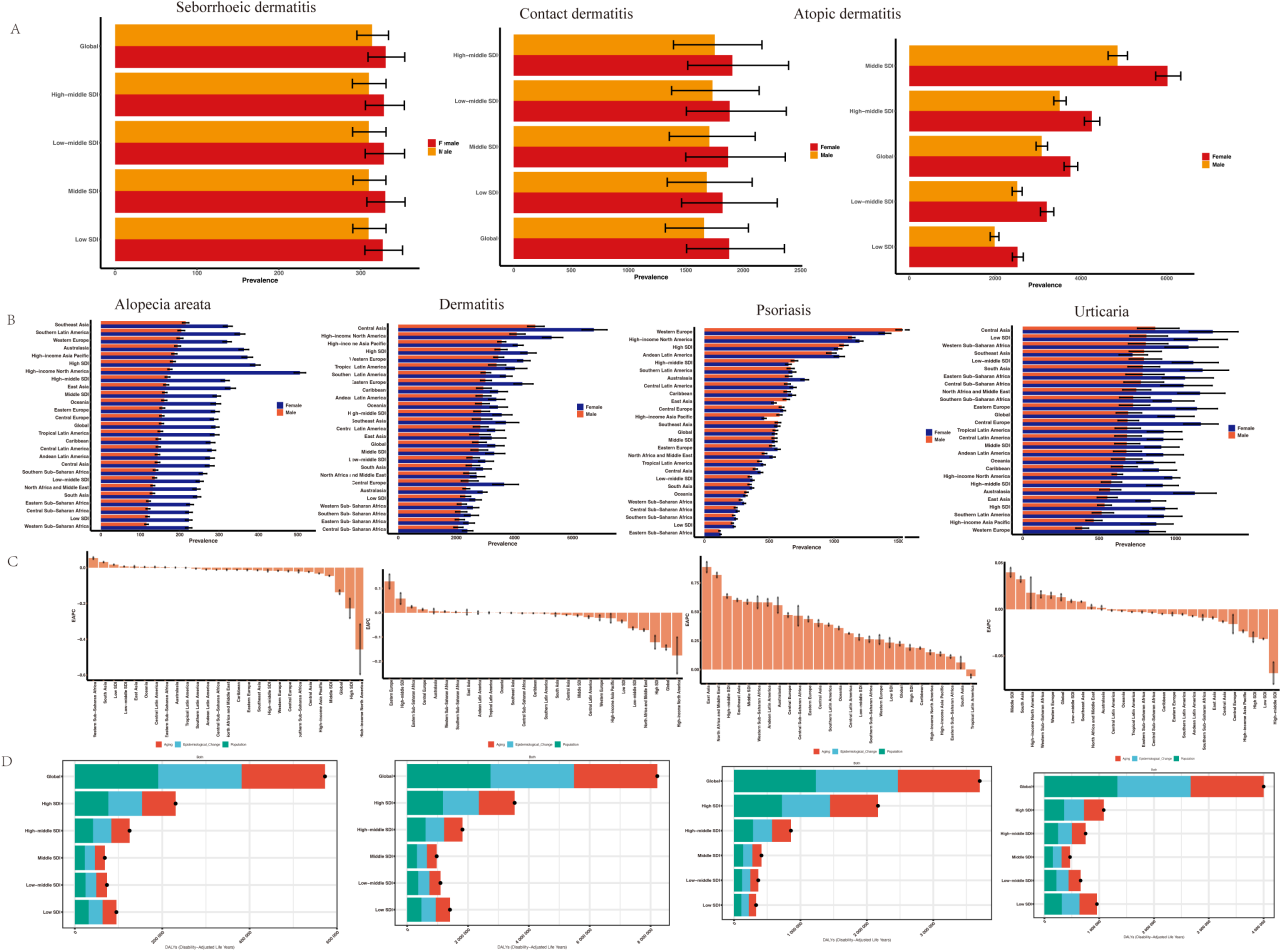
ASPR of dermatitis subtypes **(A)** and immune-related skin diseases **(B)** by sex and regions. **(C)** EAPCs in the ASPR of immune-related skin diseases. **(D)** The association between SDI and the global distribution of DALYs. EAPC, estimated annual percentage change; SDI, socio-demographic index; DALYs, disability-adjusted life years.

**Figure 2A** consistently revealed sex-based disparities in age-standardized rates of atopic dermatitis, contact dermatitis, and seborrheic dermatitis across socioeconomically diverse regions. Globally, atopic dermatitis prevalence was significantly higher in females (3753.34 per 100,000; 95% UI: 3605.98-3925.17) compared to males (3084.01 per 100,000; 95% UI: 2954.56-3220.27). The age-standardized incidence rate of contact dermatitis also demonstrated a higher rate in females (1877.11 per 100,000) than in males (1658.91 per 100,000), a difference representing a 13.15% increase. This pattern of sex-based disparity was consistent across all SDI regions, with the most pronounced difference observed in the middle-SDI region. Importantly, the non-overlapping uncertainty intervals across all comparisons confirm the statistical significance of these sex-based differences.

A consistent female predominance was observed in the age-standardized prevalence of immune-related dermatoses, including dermatitis, urticaria, alopecia areata, and psoriasis (**Figure 2B** and **Supplementary Table S2**). Globally, the age-standardized prevalence rate (ASPR) of dermatitis was higher in females (3,362.09 per 100,000) than in males (2,795.08 per 100,000), with the most pronounced difference noted in Central and Eastern Europe. The sex disparity was most prominent for urticaria and alopecia areata, moderate for dermatitis, and minimal for psoriasis. Notably, males exhibited a slightly higher ASPR than females of psoriasis in Western Europe, high-middle SDI regions, and Central Europe.

From 1991 to 2021, **Figure 2C** showed a mild global decline in alopecia areata prevalence (EAPC = -0.127; 95% CI: -0.172 to -0.083), with notable regional variation. Significant increases occurred in Western Sub-Saharan Africa and low-SDI regions, while declines were observed in High-income Asia Pacific and high-SDI regions. These trends suggest a rising burden in lower-SDI regions. The ASPR of dermatitis showed a significant declining trend (EAPC = -0.155; 95% CI: -0.222 to -0.089), though regional differences remained; Notable declines occurred in high-SDI regions (-0.151), North Africa and the Middle East (-0.068), low-middle SDI (-0.058), and low-SDI (-0.035), with the steepest drop in High-income North America, although the wide CI (-0.751 to 0.223) indicated instability. The ASPR of psoriasis increased globally, with an overall EAPC of 0.24 (95% CI: 0.12 to 0.35), while urticaria showed a minimal change, with an EAPC of 0.01 (95% CI: -0.01 to 0.03), suggesting a slightly increasing.

To reveal the causes of the increase in the burden of skin immune diseases, we used the prevalence decomposition method to break down the total changes between 1991 and 2021 into three categories: population aging, population growth, and epidemiological factors. At the global level, whether it is alopecia areata (an increase of 8.22 million), dermatitis (3.7 million), psoriasis (4 million), or urticaria, the three major contributing factors each account for approximately one-third of the overall variation, suggesting that broad structural and systemic factors—such as socioeconomic development, healthcare access, and environmental exposures—play a substantial role (**Figure 2D**).

### 3.4 Immune-related skin diseases burden in global territories

**Figure 3:**
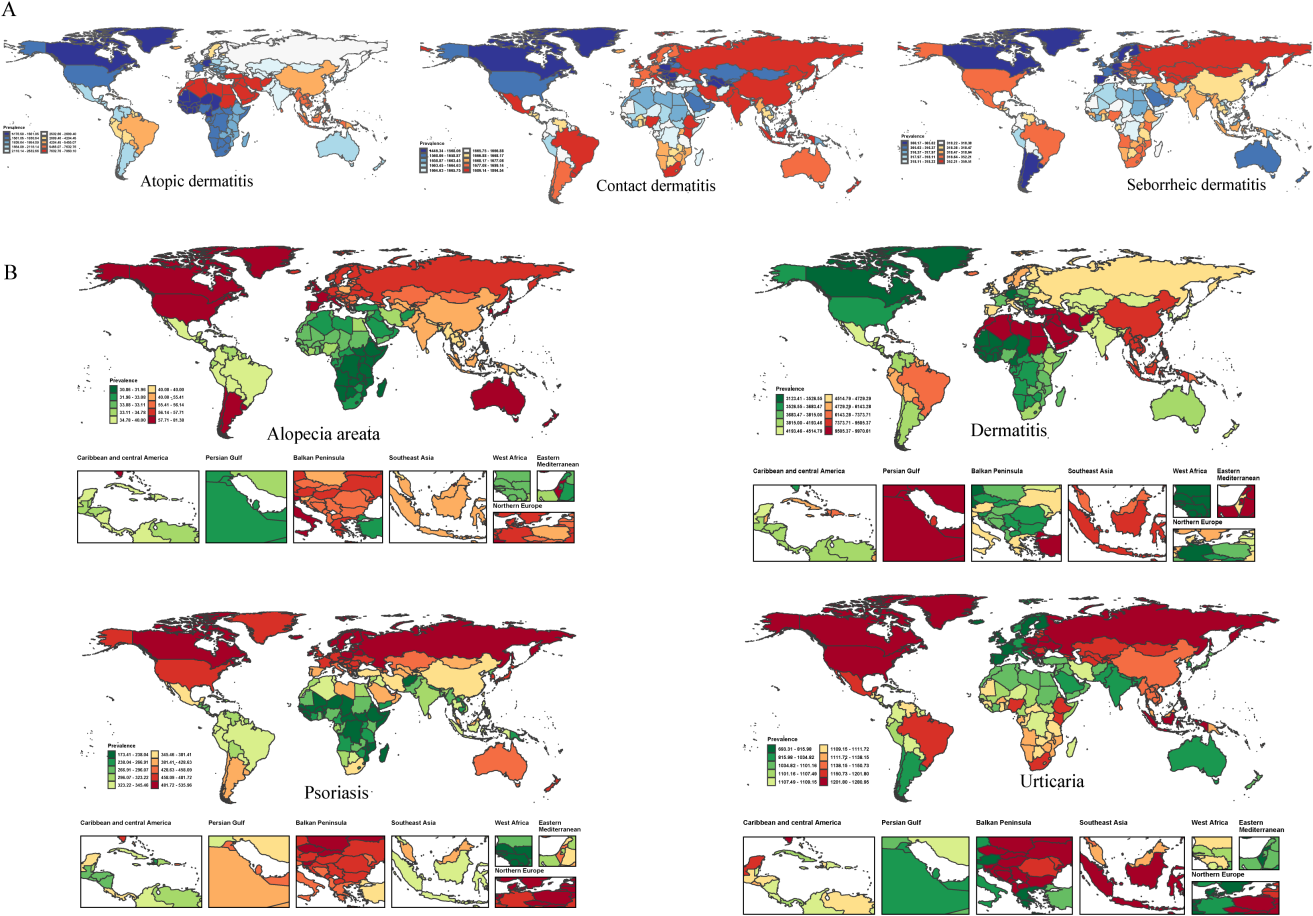
The ASR of prevalence for dermatitis subtypes **(A)** and immune-related skin diseases **(B)** in 204 countries and territories in 2021.

We analyzed the global distribution of age-standardized prevalence rates (ASPR) for immune-related skin diseases, as detailed in **Figure 3** and **Supplementary Table S3**. Dermatitis: The age-standardized prevalence rate (ASPR) was highest globally in the Islamic Republic of Iran (9871.89 [95% UI 9396.56–10405.79] per 100,000), followed by the Syrian Arab Republic (9675.04 [9112.19–10252.82]), Sudan (9658.01 [9090.49–10233.08]), Algeria (9646.43 [9078.94–10218.82]), and Turkey (9645.79 [9079.23–10215.91]). Psoriasis: The global ASPR was 354.07 (342.42–364.08) per 100,000. At the regional level, the highest rates were observed in Estonia, Lithuania, Poland, Switzerland, and Latvia. Alopecia Areata: The global ASPR was 42.89 (41.74–44.14) per 100,000. Notably, high-income regions, especially certain countries in North and South America, showed substantially higher prevalence compared to the global average. Urticaria: The global ASPR was 1094.59 (969.18–1240.42) per 100,000. The highest burden was observed in the Russian Federation, Ukraine, Poland, Canada, and Croatia, with rates significantly exceeding the global mean.

### 3.5 SDI-related differences in immune-related skin diseases burden

**Figure 4:**
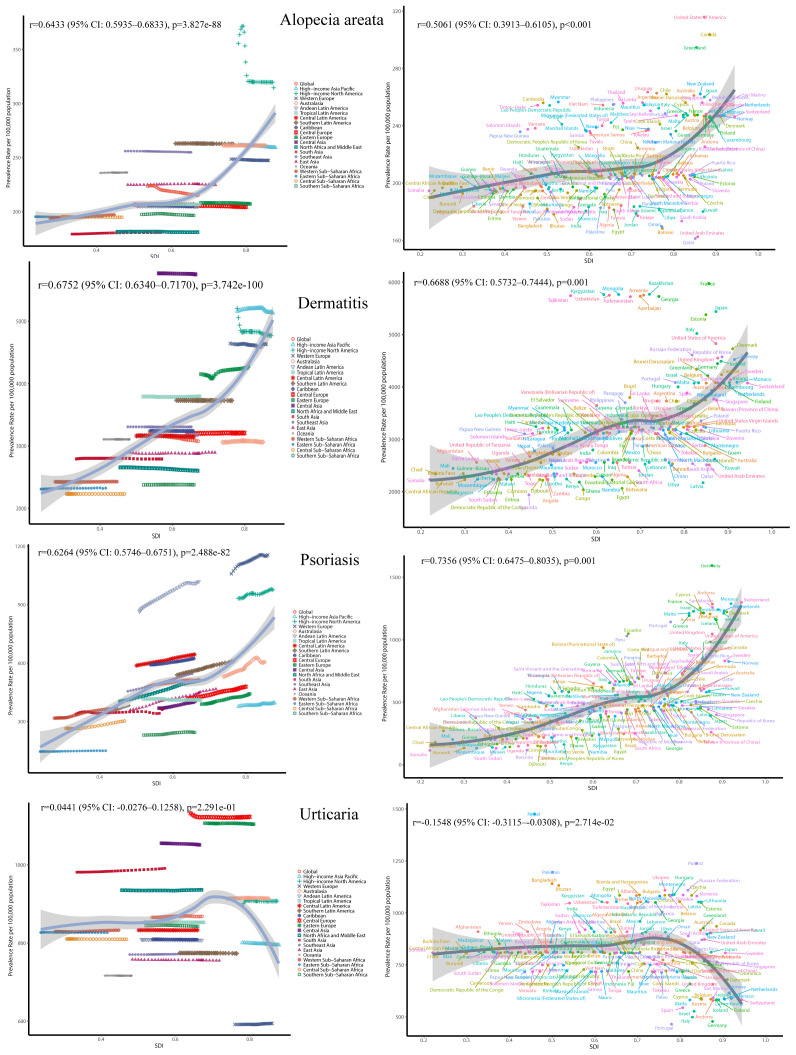
The ASR of immune-related skin diseases prevalence for 27 GBD regions and 204 countries by SDI from 1991 to 2021.

Spearman correlation analysis revealed significant positive associations between the SDI and ASPRs of alopecia areata (r = 0.6433), dermatitis (r = 0.675), and psoriasis (r = 0.6264) in **Figure 4**. This indicates that higher socioeconomic development is closely associated with higher burdens of these skin immune disorders. In contrast, urticaria showed a very weak and statistically insignificant correlation with SDI (r = 0.044, p = 0.229).

Temporally, the global ASPR of alopecia areata slightly declined from 223.61 to 215.01 per 100,000 between 1991 and 2021 (annual decrease ~0.13%), with a rise-then-fall pattern observed in high-income North America and relatively stable trends in East Asia. In contrast, both dermatitis and psoriasis showed increasing burdens in low and middle-SDI regions, while remaining stable or slightly declining in high-SDI areas, reflecting a possible disease transition driven by economic development and healthcare improvements. Notably, psoriasis exhibited a declining trend in high-SDI countries after 2010.

### 3.6 Average Annual Percent Change and prediction

**Figure 5:**
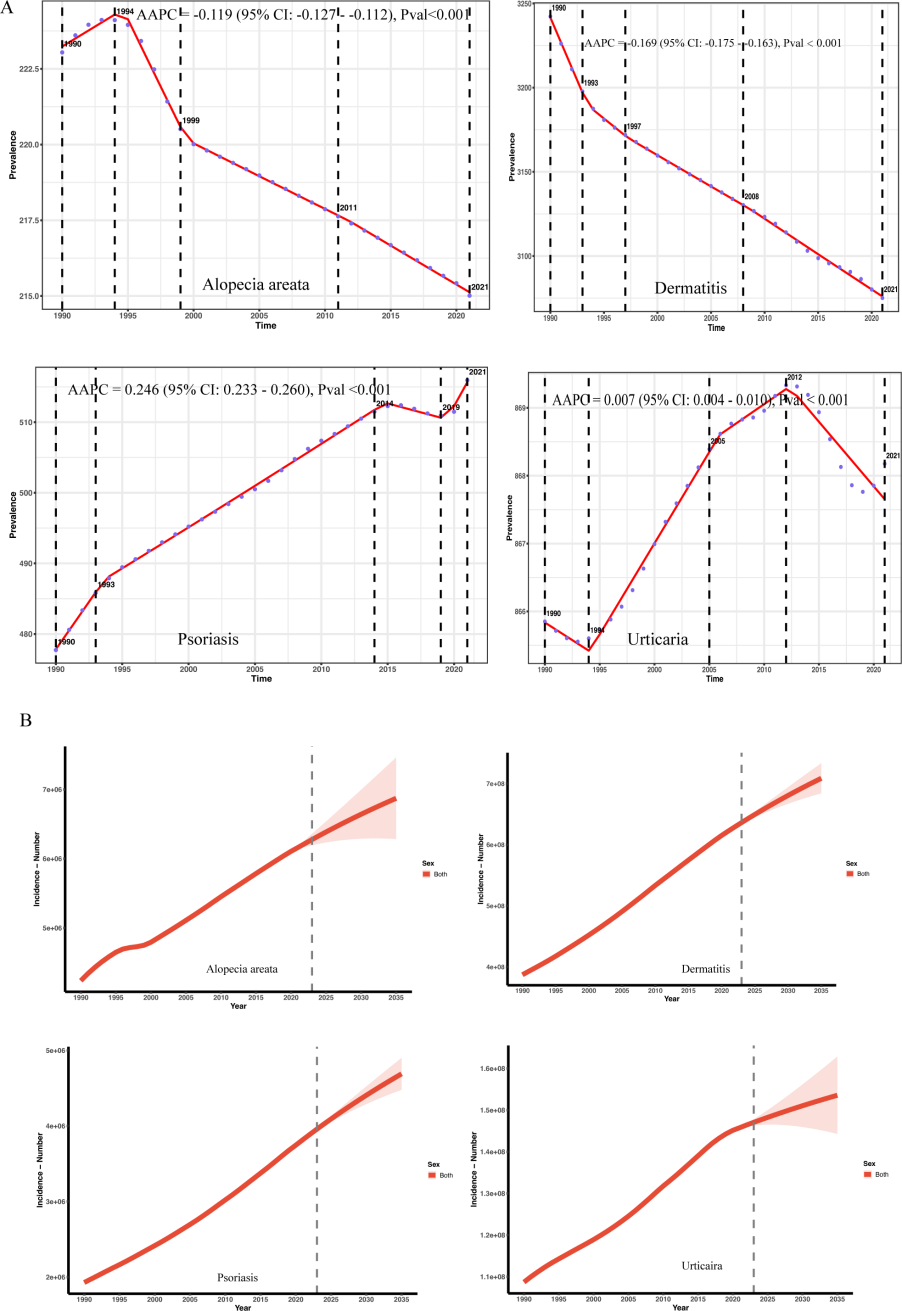
**(A)** Trends in the prevalence of immune-related skin diseases and AAPC classification from 1991 to 2021. **(B)** Trend analysis of predicted values of immune-related skin diseases to 2035.

Between 1991 and 2021, the global prevalence of dermatitis showed an overall downward trend, with an Average Annual Percent Change (AAPC) of -0.169% (95% CI: -0.175% to -0.163%, P< 0.001) in **Figure 5A**. Despite the overall downward trend, the burden of disease in low- and medium-SDI countries continued to rise, while it stabilized or slightly declined in high-SDI countries. The global prevalence of alopecia areata showed a mild downward trend. The decline has slowed down in recent years, suggesting that precise prevention and control still need to be strengthened. The global prevalence of psoriasis has increased significantly overall (AAPC = 0.246%, P< 0.001), especially in 2019–2021 and high-SDI countries have experienced a brief decline since 2010.

Analysis of Global Burden of Disease (GBD) data using a Holt damped exponential smoothing model (ETS(A,Ad,N)) reveals a continuing increase in the global prevalence of urticaria, psoriasis, alopecia areata, and dermatitis between 1990 and 2021. The magnitude of growth and damping characteristics, however, varied across these diseases. Urticaria cases increased from 108 million in 1990, with projections indicating a further rise to 153 million by 2035 (95% UI: 144–162 million) in **Figure 5B** and **Supplementary Table S4**. The annual growth rate continues to decline, consistent with the asymptotic saturation predicted by the damped trend. The model demonstrated good fit (AIC = 895.23; BIC = 904.02; MAPE = 9.8%; RMSE = 174,100). The damping term suggests a gradual attenuation of growth momentum, aligning with an epidemiological phase of stabilizing population structure. Psoriasis cases increased from 1.928 million in 1990 to 3.818 million in 2021, reflecting an average annual growth rate of approximately 2.9%. Model projections indicate a number of 4.689 million cases by 2035, although the growth rate is expected to decrease to 1.7% between 2022 and 2035. The damping trend was significant (damped = TRUE), and the model exhibited good fit (AIC = 643.39; BIC = 652.19; MAPE = 1.00%; RMSE = 3403.77). The significantly widened 2035 forecast interval (95% UI: 4.479–4.899 million) indicates increased uncertainty in long-term forecasts and should be interpreted with caution, considering potential influences from socioeconomic and policy factors. Global alopecia areata prevalence increased from 4.24 million in 1990 to 6.16 million in 2021, with a gradually slowing rate of growth. Forecasts suggest a slow increase to 6.87 million between 2022 and 2035 (95% UI: 6.29–7.46 million). While the model fit was excellent (AIC = 765.81; BIC = 774.60; MAPE = 31%; RMSE = 23048.18), its predictive power is limited, and extrapolated projections should be interpreted cautiously. The number of individuals with dermatitis is projected to reach 709 million by 2035. Although the overall trend continues upward, the rate of growth is gradually slowing, consistent with the non-exponential growth characteristic of a damped model. The widening forecast range (95% UI: 684–733 million) reflects the accumulation of long-term uncertainty. The model demonstrated excellent performance (MAPE = 6.7%; RMSE = 398,000; AIC = 948.1; BIC = 956.9). The damping mechanism effectively mitigated exponential growth, consistent with established biological and demographic principles.

## 4 Discussion

Based on the systematic analysis done by the GBD 2021, this study provides up-to-date insights into the global, regional, and national burden of immune-related skin diseases from 1990 to 2021, and forecasted estimates of disease burden to 2035 for the first time. The analysis showed that different diseases and regions showed significant gender, age, and socioeconomic-related differences. Disease burdens in low-SDI regions may be underestimated due to underdiagnosis and limited surveillance (29), While the higher burden of skin immune diseases in high-SDI regions may partly reflect greater diagnostic capacity.

Alopecia areata is the most prevalent autoimmune disorder and the second most prevalent hair loss disorder after androgenetic alopecia. Alopecia areata showed a female-male ratio of 1.87:1, which is further supported by ten hospital-based studies worldwide reporting a female predominance with ratios ranging from 2.6:1 to 1.2:1 (30–35). This cause may be related to gender-related immune mechanisms, where the immune system erroneously targets hair follicles, leading to patchy hair loss. The incidence of alopecia areata based on self-reported data is relatively low, which may lead to an underestimation of its true global burden (36). Emerging evidence suggests that the pathogenesis of immune-related skin diseases may be linked to dysbiosis of the microbiota (37). Certain ethnic groups have a higher prevalence of alopecia areata in women; for example, Hispanic, Asian/Pacific Islander, and African American women have significantly higher age-and sex-adjusted prevalence rates than non-Hispanic white women (38). Our data indicate that Spain’s ASPR of 57.71 (95% UI: 55.94–59.70) exceeds the global average of 42.87, a finding that aligns with our overall research observations. The pathogenesis of alopecia areata in women may involve both immune attacks on hair follicles triggered by hormonal fluctuations (e.g., estrogen, progesterone) (39) and molecular pathways associated with dysregulated activity of localized aromatase and 5α-reductase (40). Women are more likely to suffer from psychological disorders (such as anxiety and depression) due to hair loss (41). Numerous studies have underscored the psychological health challenges frequently observed in patients with alopecia areata (42, 43). The increased prevalence of alopecia areata in women may be attributable to gender-related immune mechanisms (44, 45), ethnic and age-related predispositions (46), and potentially external physical factors. These observations highlight the multifactorial etiology of the disease, and further investigation is warranted to elucidate these potential associations. Notably, the prevalence of dermatitis, psoriasis, and alopecia areata all increased with higher SDI levels. These disparities may be partly attributed to differences in socioeconomic development, environmental exposures, and healthcare accessibility.

Psoriasis is a systemic inflammatory condition with effects extending beyond the skin. Our findings reveal notable geographical variations in both the prevalence and disease burden of psoriasis (47, 48). Psoriasis accounted for a disproportionately high proportion of total DALYs – approximately 9–12% – compared to its contribution to the overall incidence rate (<1%). The significant long-term disability associated with psoriasis likely stems from its chronic nature, comorbidities, and substantial reduction in quality of life. Its chronic inflammatory state can lead to joint involvement, causing structural damage and functional limitation, and can affect other organ systems (49). Psoriasis is frequently associated with comorbidities such as obesity, diabetes, and hypertension, collectively accelerating cardiovascular disease progression (50). Notably, the risk of major adverse cardiovascular events, including myocardial infarction and peripheral vascular disease, correlates positively with psoriasis severity, as measured by psoriasis area and severity index scores (51). The disability associated with psoriasis arises from the complex interplay of biological, psychological, and social factors, necessitating multidisciplinary management to improve long-term outcomes (52). Our study further reveals a significant increase in the global prevalence of psoriasis between 2019 and 2021, a resurgence possibly influenced by the COVID-19 pandemic (53). The COVID-19 pandemic highlighted the difficulties in managing immunosuppression or immunomodulation, treatment modification, and the initiation of new therapies in patients with conditions such as psoriasis, atopic dermatitis, and hidradenitis suppurativa (54). Emerging evidence suggests SARS-CoV-2 infection may exacerbate psoriasis, supported by elevated plasma inflammatory cytokines (e.g., granulocyte-colony stimulating factor, tumor necrosis factor-alpha) correlating with disease severity (55, 56). These findings underscore the importance of early identification and comprehensive management of psoriasis, not only as a dermatologic disorder but as a systemic disease with substantial global health implications.

Urticaria, commonly known as hives, is a prevalent dermatological condition characterized by transient, pruritic wheals that can significantly impair quality of life. Our research suggested urticaria exhibits a peak prevalence in early childhood. Children under five years, particularly girls, show the most rapid increase in urticaria prevalence and a significantly rising absolute disease burden. The disease burden, as measured by the DALY rate, is higher in female children than in males (57). Acute urticaria is far more common than chronic urticaria in the pediatric population, with approximately 20% of children experiencing acute episodes (58). While urticaria in children under ten is primarily acute and often self-limiting, vigilance regarding long-term management and quality of life remains essential for the minority of cases progressing to chronicity. Despite this stability, the disease’s impact varies among different regions and populations. Similarly, in South Africa, a review of data from two tertiary referral centers in Cape Town provided insights into the local epidemiology of urticaria (59). Their study emphasizes that while the overall prevalence may be similar to global estimates, regional factors influence disease presentation and healthcare utilization. The epidemiology of urticaria in children has been examined through an analysis of data from insured German individuals under 18 years of age (60). Their retrospective study demonstrated that pediatric urticaria is a significant health issue, with patterns in incidence and diagnosis. Furthermore, the epidemiology of urticaria in older adults has been explored by Patruno, highlighting unique clinical considerations and management challenges in this age group (61). Emerging research also points to increasing recognition of urticaria in specific populations, such as pregnant women and the elderly. Epidemiological considerations in pediatric, pregnant, and lactating populations have been highlighted in recent research (62). These insights are essential for developing age-appropriate management strategies.

In our research, atopic dermatitis, contact dermatitis, and seborrheic dermatitis all showed consistently higher prevalence and incidence rates in females than in males across all SDI regions. Multiple studies have reported that the prevalence of atopic dermatitis in adult females is significantly higher than in males (63, 64). Potential food allergens present in cosmetic products may serve as triggers for atopic dermatitis development (65). Although contact dermatitis had the highest incidence worldwide, atopic dermatitis contributed the most to the global disease burden, indicating that its impact on disability and quality of life is disproportionately greater relative to its incidence. Although the global incidence of atopic dermatitis is lower than that of contact dermatitis, its chronic, relapsing nature, and systemic effects—such as Th2 immune dysregulation and associated comorbidities—contribute to a more substantial long-term cumulative burden (66). This is further evidenced by the fact that the growth rate of AD-related DALYs outpaces its incidence growth, highlighting its disproportionate disease burden (67). Moreover, the economic impact of atopic dermatitis encompasses both direct treatment costs and indirect productivity losses, with a particularly pronounced burden in middle-income countries (68).

Early investigations that emphasized the widespread nature and rising prevalence of atopic dermatitis have been substantiated by our studies (69). Subsequent studies have emphasized the rising incidence of atopic dermatitis while also exploring its potential causes, suggesting that both environmental and genetic factors play crucial roles in disease development (70). Recent data showed a rising prevalence of atopic dermatitis, affecting 10–20% of U.S. children, with about 10.7% newly diagnosed each year (71).

Females are the predominant group among patients with facial contact dermatitis (71.06% vs. 28.94%) and exhibit a higher rate of positive patch test reactions (72). This sex disparity is closely linked to behavioral exposure patterns; adolescent females (aged 12–18) have significantly greater exposure to cosmetics and metal accessories (e.g., earrings), resulting in a 2.9-fold increased risk of cosmetic-related contact dermatitis compared to males (OR = 2.9) (73). Moreover, during the COVID-19 pandemic, females were more susceptible to facial dermatitis triggered by prolonged mask wear, attributed to friction and a localized humid environment, with common clinical manifestations including bilateral cheek itching and erythema (74). Meanwhile, the high prevalence but relatively low incidence of contact dermatitis suggests that the condition tends to be chronic or recurrent, with a prolonged disease duration and incomplete recovery in many patients. This pattern implies that once contact dermatitis develops, it often persists or relapses, highlighting the need for long-term prevention and management strategies. Occupational exposure—such as to medical equipment and hairdressing chemicals—or contact with essential daily items like nickel-containing products makes complete allergen avoidance unrealistic, leading to recurrent episodes of the disease (75). Data from the Finnish national registry indicate that contact dermatitis can result in prolonged sick leave and loss of work capacity, underscoring the chronic course and substantial socioeconomic burden of the condition (76).

During the COVID-19 pandemic, a study reported that among 228 patients, at least one hair disorder was identified, with seborrheic dermatitis being the third most common condition, following telogen effluvium and hair graying (77). Further research is warranted to elucidate the relationship between seborrheic dermatitis and COVID-19. Another study demonstrated that the detection rate of seborrheic dermatitis was significantly higher in females than in males (p = 0.015), and that female patients tended to develop the disease at an earlier age (p = 0.048) (78). Our research further supports this conclusion.

## 5 Limitation

This study has several limitations. First, the dataset lacked detail on disease subtypes and severity, preventing analyses of acute versus chronic forms. Second, underdiagnosed, self-limiting, or mild cases may be underestimated, particularly in regions with limited healthcare access. Third, sparse or inconsistent data from underrepresented regions could affect estimate reliability. Fourth, improvements in diagnostics and reporting over time may confound temporal trends. Fifth, aggregated data precluded assessment of individual-level factors, limiting causal inferences. Additionally, the GBD database does not capture natural remission, potentially overestimating prevalence. Additionally, the GBD database does not capture natural remission, potentially overestimating prevalence. In addition, the projections presented in this study are based on the Holt’s damped trend exponential smoothing model (ETS(A,Ad,N)), which relies on historical trends of incidence and prevalence. These forecasts assume a continuation of current patterns and do not explicitly account for potential changes in healthcare access, novel interventions, policy shifts, or unexpected events (e.g., pandemics). Therefore, while the model provides reasonable short- to medium-term estimates, the predictions should be interpreted as trend-based scenarios rather than precise forecasts. Uncertainty intervals are provided to reflect potential variability, but caution is advised when extrapolating beyond the observed data range.

## Data Availability

The original contributions presented in the study are included in the article/**Supplementary Material**. Further inquiries can be directed to the corresponding authors.
